# Artificial Red Blood Cells as Potential Photosensitizers in Dye Laser Treatment Against Port-Wine Stains

**DOI:** 10.3390/jfb8020014

**Published:** 2017-04-13

**Authors:** Naoaki Rikihisa, Shoji Watanabe, Yoshiaki Saito, Hiromi Sakai

**Affiliations:** 1 Chiba Rosai Hospital, 1-16 Tatsumidaihigashi, Ichihara, Chiba 290003, Japan; 2 Saitama Children’s Medical Center, 1-2 Shintoshin Chuo-ku Saitama, Saitama 330877, Japan; watanabe.shoji@scmc.pref.saitama.jp; 3 The Laboratory of Pathology, Hatano Research Institute, Food and Drug Safety Center, 729-5 Ochiai Hatano, Kanagawa 2578523, Japan; saito.y@fdsc.or.jp; 4 Department of Chemistry, Nara Medical University, 840 Shijo-cho, Kashihara 6340813, Japan; hirosakai@naramed-u.ac.jp

**Keywords:** artificial blood cells, hemoglobin vesicle, capillary malformation, port-wine stain, laser treatment, light therapy equipment, photosensitizer, chromophore

## Abstract

We suggest a novel method that uses artificial blood cells (hemoglobin vesicles, Hb-Vs) as photosensitizers in dye laser treatment (at 595-nm wavelength) for port-wine stains (i.e., capillary malformations presenting as red birthmarks) based on the results of animal experiments. As compared with human red blood cells, Hb-Vs have the same absorbance of 595 nm wavelength light and produce the same level of heat following dye laser irradiation. Small sized Hb-Vs (250 nm) distribute in the plasma phase in blood and tend to flow in the marginal zone of microvessels. Intravenous injections of Hb-Vs caused the dilatation of microvessels, and dye laser treatment with Hb-Vs destroyed the vessel wall effectively. Following the intravenous injection of Hb-Vs, the microvessels contained more Hb that absorbed laser photons and produced heat. This extra Hb tended to flow near the endothelial cells, which were the target of the laser treatment. These attributes of Hb-Vs will potentially contribute to enhancing the efficacy of dye laser treatment for port-wine stains. Hemoglobin is a type of porphyrin. Thus, our proposed treatment may have aspects of photodynamic therapy using porphyrin that leads to a cytotoxicity effect by active oxygen.

## 1. Introduction

Artificial red blood cells have been developed as an alternative to blood transfusions in order to maintain systemic circulation and oxygenation during acute hemorrhage. We suggest a change of this recognition of the sophisticated hemoglobin vesicle (Hb-V) from oxygen carriers to photosensitizers in vessels such as photodynamic therapy. We investigated the use of Hb-Vs as photosensitizers in the dye laser treatment of port-wine stains (PWSs). We summarize our results of in vivo and in vitro experiments to measure the aptitude of Hb-V as a photosensitizer, and discuss the advantages of this method [1,2,3].

## 2. Pathology of Port-Wine Stains

**Figure 1:**
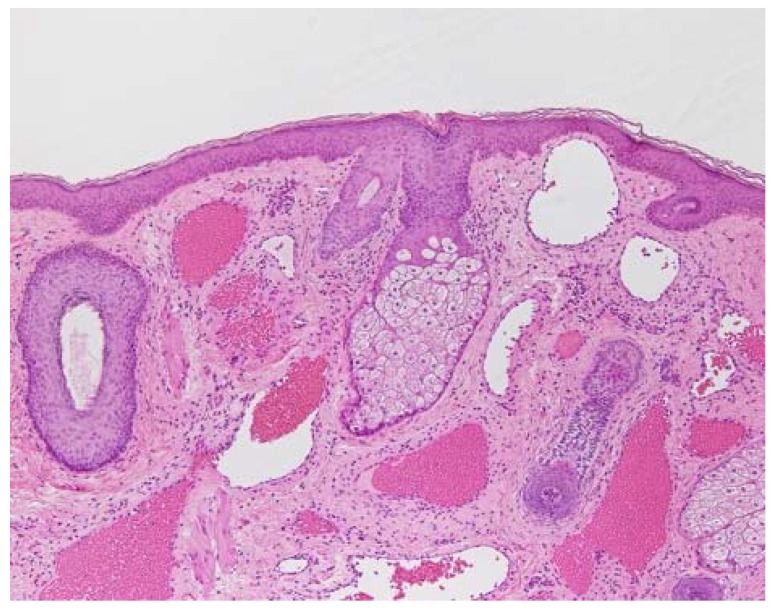
Histologic appearance of the facial port-wine stain of a 40-year-old man. (Hematoxylin staining; ×100). The dilated capillaries are filled with red blood cells. The vessel walls are thin and are lined by flat mature endothelial cells.

**Figure 2:**
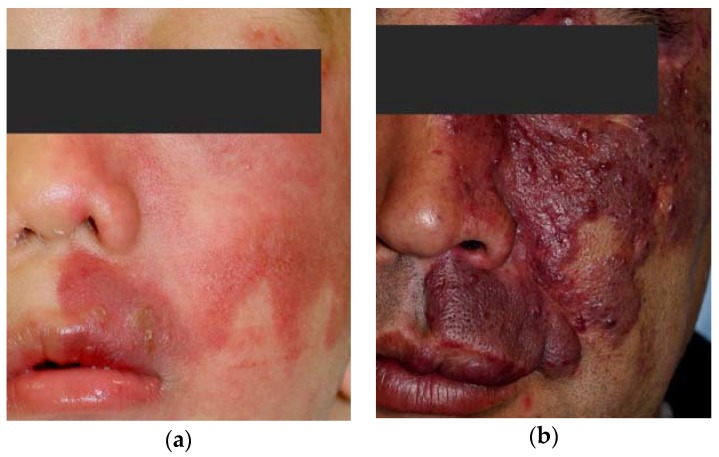
(**a**) Clinical appearance of a port-wine stain on a 3-month-old child. Port-wine stains are present at birth. They are flat and sharply demarcated; (**b**) Clinical appearance of a port-wine stain on the face of a 40-year-old man. The color of the port-wine stain gradually darkens to a shade of red during young adulthood and then to deep purple during middle age. In addition, the surface of the port-wine stain becomes raised with aging and studded with nodular lesions.

Some birthmarks have the appearance of the skin having been discolored by wine, hence the name “port-wine stain” PWSs are common capillary malformations characterized on histological examination by dysfunctional dilated microvessels in the normal dermis (Figure 1). Their incidence is 0.3%, and they are more prevalent in boys [4,5,6]. Capillary malformations on the face are commonly sub-classified as lateral (PWSs) (Figure 2a) and medial (also known as “salmon patches”). Lateral PWSs on the face always persist, whereas medial lesions usually become lighter and some disappear, particularly those on the mid-face [5]. PWSs generally grow in proportion to body size, gradually darkening to a shade of red during young adulthood and then to a deep purple in middle age. Aging also raises the surface of PWSs, which becomes studded with nodular lesions (Figure 2b) [4,5,6].

## 3. PWSs Treatment with Light Therapy Equipment

**Figure 3:**
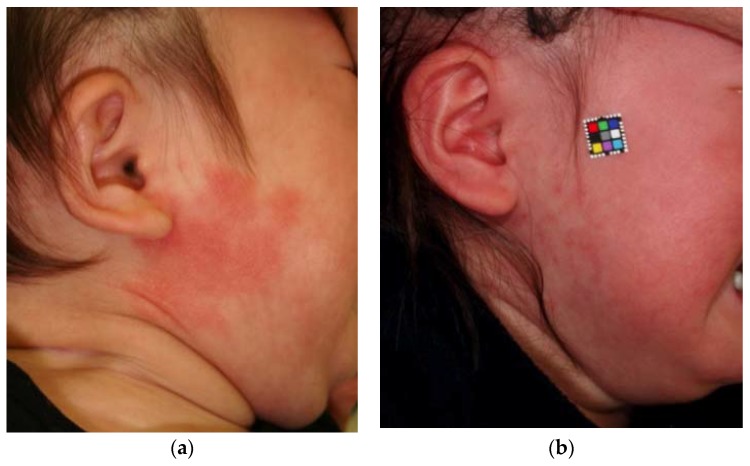
(**a**) An 8-month-old child with a port-wine stain on her right cheek before treatment; (**b**) Clinical appearance of the same case after two sessions of variable long-pulse pulsed dye laser treatment in four months. The treatments were successful in this case.

Since the 1980s, the flashlamp-pumped pulsed dye laser (PDL) has been considered the best laser system for the treatment of PWSs (Figure 3) [5,7].

**Scheme 1:**
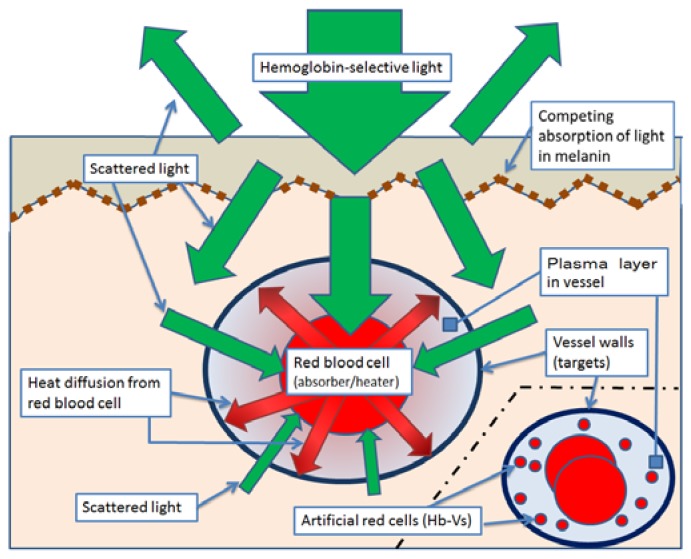
Heating of microvessels containing red blood cells and artificial red cells by light therapy equipment.

Laser treatment for PWSs is based on selective photothermolysis, in which vascular-selective laser light is absorbed by the hemoglobin (Hb) in red blood cells (RBCs) (the absorbers and heaters); this produces heat, which results in photocoagulation and the aggregation of RBCs and ultimately in necrosis of the endothelial cells (the targets) (Scheme 1) [7,8,9]. Under appropriate laser parameters, the thermal energy from photothermolysis is released selectively into the target tissue without exposing the surrounding tissues such as the dermis and nerves [7,8,9].

Various vascular-selective lasers and light illumination can be used, depending on the condition and position of the affected skin and vessels. These include pulsed dye lasers [7], Alexandrite lasers, neodymium-doped yttrium aluminum garnet (Nd: YAG) lasers, and intense pulsed light using a xenon flashlamp. Nd: YAG lasers are recommended for treating lesions on lips. Alexandrite and Nd: YAG lasers are recommended for treating hypertrophied PWSs or resistant lesions in which the affected vessels are in deep layers, because these types of vascular-selective lasers have a high depth of penetration [7].

## 4. Effect and Limit of PWS Treatment with Light Therapy Equipment

The aim of these treatments is to change the skin color of the lesion to that of healthy skin by destroying the affected microvessels in the dermis without injuring the surrounding tissue. Such treatments can reduce the severity of complications, including cutaneous hypertrophy, disfigurement of the normal tissue architecture, and psychosocial morbidity [5,7]. The use of dynamic skin surface cooling, which reduces the risk of epidermal damage and minimizes the pain of treatment, has allowed the application of more effective, higher energy fluence. Devices modified to enable longer pulse widths, longer wavelengths, and larger spot sizes have improved the efficacy of PDL in treating lesions sited in deeper layers or those which contain vessels of a larger diameter [5,7,10].

Despite the improvements, only one-fifth of PWSs clear completely, although the color of most capillary malformations improves significantly with treatment [5]. In our experience of treating 708 cases of PWSs, 69% of the patients received one or two sessions of laser irradiation, 20% received three to five sessions, and 11% received six or more sessions. Our data show that 10%–20% of the PWSs remained resistant to laser treatment.

These laser treatment-resistant PWSs show specific histological characteristics. Some vessels may be too large or the blood flow may be too fast, thus preventing the generation of sufficient energy to damage the vessels irreversibly [11]. It is likely that short pulse-width pulsed dye lasers do not destroy large, partially coagulated vessels and these then regenerate after treatment [11]. The coagulation of deeper vessels was not observed in PWSs with multiple vascular layers because of the absorption of laser light in the more superficial vessels [11]. In addition, vessels with a diameter <30 μm are resistant to PDL treatment because they do not contain sufficient RBCs and are thus poor at absorbing the laser radiation and heating the vessel [11].

In Japan, dye lasers have been used in trial treatments for PWSs. Before the laser irradiation, the redness of the affected skin was emphasized by prior treatment using methods such as infrared ray heating or cupping therapy [12], in which local suction was created on the skin. However, the practical effectiveness of these treatments has not been verified. Other clinical trials have been performed with the aim of improving the outcome of PWS treatment. Antiangiogenic agents such as topical rapamycin have been applied after laser treatment to prevent the recurrence of the affected vessels [7]. Long-term follow-up is required to assess the safety of this new treatment. Photodynamic therapy, which combines a chemical photosensitizer, laser irradiation, and tissue oxygen, represents an alternative treatment option for PWSs. In this process, the chemical photosensitizer absorbs laser light and produces reactive oxygen species, which form free radicals that destroy the endothelial cells of the vessel wall. This treatment is less commonly used as the intravenous injection of the photosensitizer typically results in photosensitivity persisting for days to weeks [7,13].

## 5. Break Through the Limits of Laser Treatment Against PWS with Hb-Vs

Hb-Vs are cellular particles that encapsulate a purified and concentrated Hb solution within a phospholipid bilayer membrane, acting as artificial red blood cells. The final Hb concentration was adjusted to 10 g/dL [14,15,16]. The diameter of an Hb-V is 1/40 the diameter of a human RBC, and so after an injection the Hb-Vs flow among the RBCs in the microvessels [3,17]. The hemoglobin vesicle injection increases the hemoglobin concentration in the microvessels. Based on the principle of treating port-wine stains with a dye laser, intravenous injection of artificial red cells (Hb-Vs) immediately before laser treatment might improve the clinical outcome of the therapy.

### 5.1. Observation of Artificial Red Blood Cells in Microvessels and Microtubes

**Figure 4:**
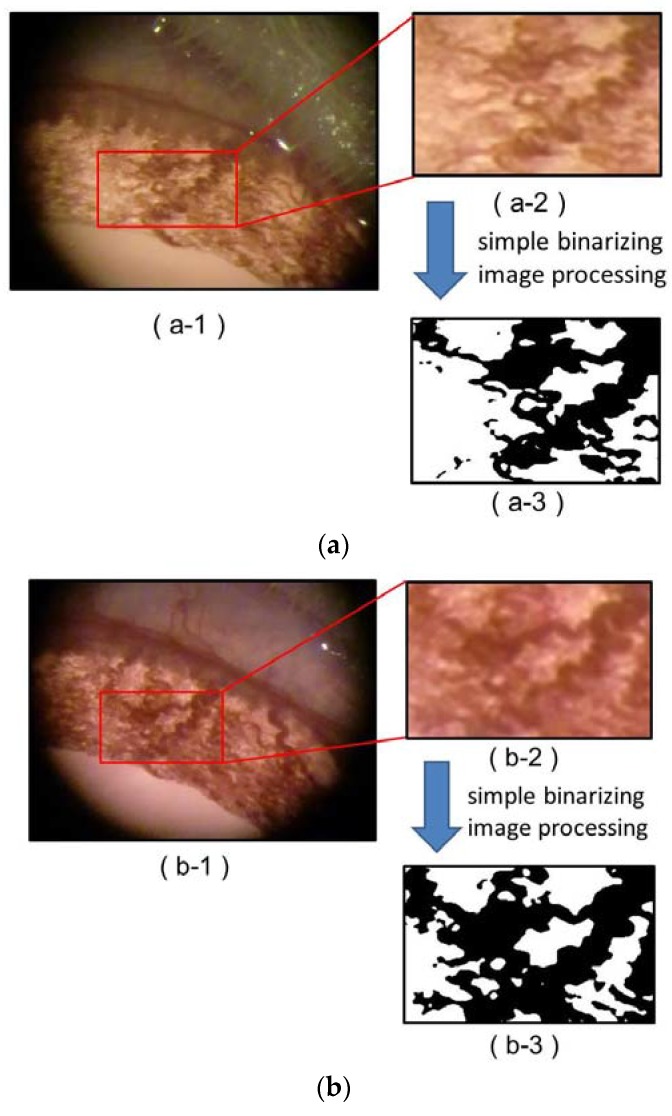
Microvessels of a rat’s iris viewed under a magnifying glass before and after an injection of Hb-Vs [1] (**a-1**) Before the injection; (**a-2**) The specific area in the rat’s iris before the injection; (**a-3**) Two-dimensional changes in the microvessels were analyzed by area extraction using simple binarizing image processing (Image J, National Institutes of Health, Maryland, USA); (**b-1**) After the injection of Hb-Vs, showing a marked expansion in the microvessels, with the microvessel area in the bird’s-eye view increasing by 20%; (**b-2**) The specific area after the injection; (**b-3**) Two-dimensional changes in the microvessels were analyzed by area extraction using simple binarizing image processing.

The microvessels of a rat iris are visible with a magnifying glass through the transparent cornea; this allows the observation of dynamic changes under minimally invasive conditions. Following the injection of Hb-Vs (at a dose of one-tenth of the circulating blood volume), the microvessels were observed to expand and to become markedly dense. The specific area of the microvessels in a bird’s-eye view increased by 20% after the injection (Figure 4) [1].

**Figure 5:**
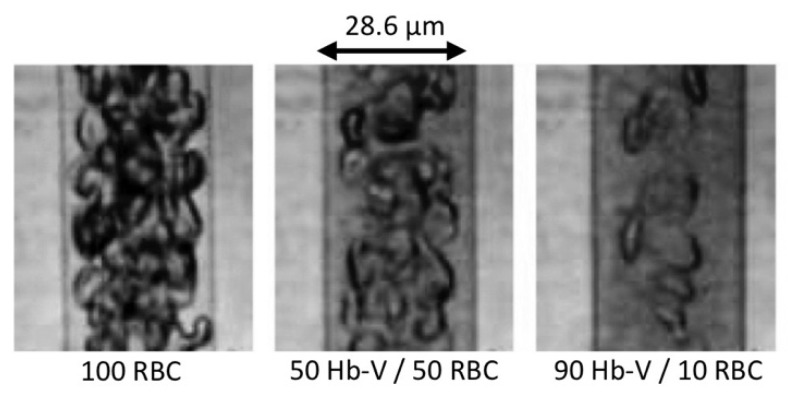
Flow patterns of red blood cells (RBCs) mixed with Hb-Vs suspended in human serum albumin in a narrow tube (28.6 µm in diameter). The Hb-V solutions were mixed with the RBC suspension at volume ratios (Hb-V/RBC) of 0:100, 50:50 and 90:10. The Hb-V particles were homogeneously dispersed in the suspension medium but tended to become distributed in the marginal zone of the flow. The thickness of the RBC-free layer increased with increasing amounts of Hb-V, with the RBC-free phase becoming darker and semitransparent, indicating the presence of Hb-Vs. Hb concentration, 10 g/dl; centerline flow velocity, 1 mm/s [3,16].

In microvessels, the RBCs tend to become concentrated towards the vessel center, leaving a significant layer free of RBCs near the vessel walls. A large quantity of small-size Hb-Vs would flow through this RBC-free layer because they are small and are forced away from the vessel center (Figure 5) [3,18]. The Fåhræus effect is the phenomenon where, in blood vessels with diameters <500 µm, the hematocrit decreases with the capillary diameter [19]. Increasing the Hb concentration using Hb-Vs would be expected to result in a more efficient conversion of laser light into thermal energy by an increase in the number of heaters for the photothermolysis treatment in the microvessels (Scheme 1) [7,8,9]. Given the results of rheological studies of Hb-Vs, it appears to be possible to increase Hb density (and therefore their absorption and heating effects), especially near the vascular endothelial cells (the targets) of smaller microvessels by the administration of Hb-Vs. This effect could potentially improve the treatment for PWSs.

### 5.2. The Effects of Dye Laser Irradiation on Hb-Vs

**Figure 6:**
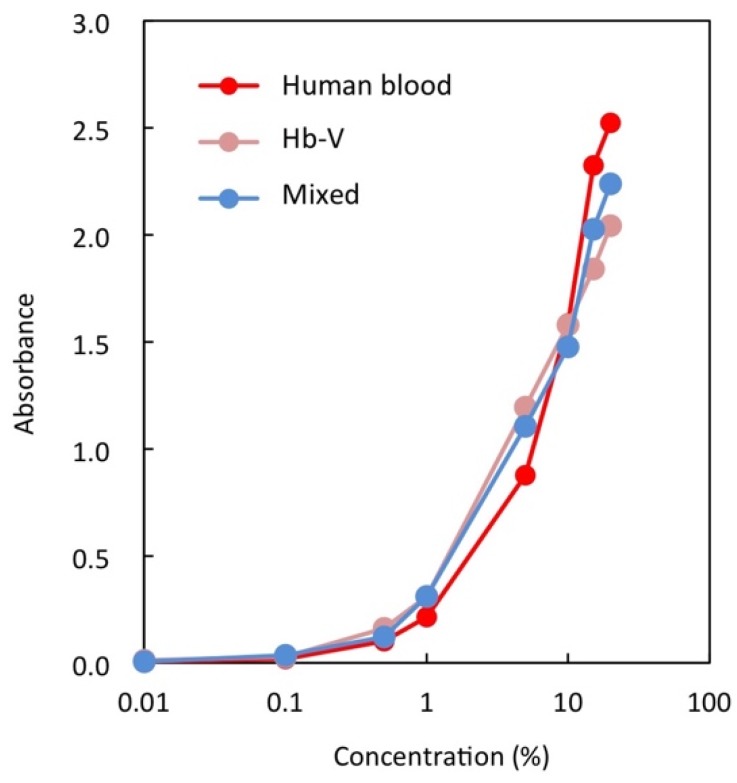
Absorbance at 595 nm in heparinized human blood dispersed in normal saline, Hb-Vs dispersed in normal saline, and a 1:1 mixture of blood and Hb-Vs in saline [1]. Each 100% concentration sample contained 10 g/dL Hb. Ultrospec 3000 UV/Visible Spectrophotometer (Pharmacia Biotech, New Jersey, USA; wavelength accuracy ± 1 nm; photometric accuracy ± 0.5% or ±0.003–3.000 A, whichever was the greater; light path 10 mm) was used for absorbance measurement.

We characterized in vitro the basic photoreactions and photosensitizer effects of Hb-Vs irradiated by a dye laser with a wavelength of 595 nm in comparison with those of human RBCs. To confirm that the photosensitizer effects of Hb-V and blood mixed with Hb-V were the same as those of blood at the same Hb concentration (10 g/dL), we mixed blood and Hb-V at a ratio of 1:1 of Hb-V to Hb. We analyzed samples in three groups: heparinized human blood dispersed in normal saline (the hBlood group); Hb-Vs dispersed in normal saline (the Hb-V group); and the 1:1 mixture of blood and Hb-V in saline (the Mixed group). Absorbance at 595 nm at various concentrations of Hb was determined using a spectrophotometer. The hBlood, Hb-V and Mixed group samples all showed the same absorbance at 595 nm at all concentrations of Hb (Figure 6) [1].

To check the temperature increase with laser irradiation, we irradiated 2 mL samples from each group at an Hb concentration of 10 g/dL using constant laser parameters (pulse number, 2; pulse width, 0.5 ms; fluence, 10.1 J/cm^2^, with V-star, Cynosure; wavelength, 595 nm; pulse width, 0.5–40 ms). We used a securing device to maintain a fixed distance between the samples and the laser handset. The temperature of each sample before and after irradiation was measured using a high-resolution digital thermometer (ThermoProbe TL1-R12, ThermoProbe, Inc., Mississippi, USA; resolution, 0.001 °C; tolerance, ±0.04 °C). After the dye laser irradiation test, fluid temperatures increased by 0.345 °C in the human blood fluid, by 0.304 °C in the Hb-V fluid, by 0.365 °C in the Mixture fluid (1:1). We used a securing device to maintain a fixed distance between the samples and the laser handset. The three sample groups showed the same change in temperature after irradiation with the dye laser, with Hb concentration maintained at 10 g/dL [1].

### 5.3. Changes in Chicken Wattle Microvessels with Dye Laser Irradiation after Hb-V Intravenous Injections

We used chicken wattle as an animal model of PWSs. Its histologic anatomy has a state that is analogous to PWSs in terms of the rich vascularity of the dermis, and the skin of the wattle contains only a low level of melanin, which has benefits for laser irradiation [20,21].

We divided the animals into three groups, irradiated at different fluences. These groups were then divided into two subgroups: control animals which did not receive Hb-V injections and intervention animals that received an intravenous injection of a bolus of Hb-V at a dose of one-tenth of the circulating blood volume. Thirty minutes after the Hb-Vs intravenous injection, we irradiated 7-mm-diameter areas of the wattle at the three fluences (4 J/cm^2^, 5 J/cm^2^, and 6 J/cm^2^) at a fixed pulse width of 10 ms. The clinical endpoint goal during laser therapy was a transient gray to blue discoloration of the skin that evolved into purpura. We adopted these parameters of laser output because they were the borderline values producing skin discoloration in previous experiments [7].

For each irradiated area, we evaluated 20 vessels with an average diameter of ≥25 nm, scoring them according to the scoring system in Table 1 [1]. Chi-square tests were used to determine the effect of the laser irradiation on the scores of the 240 irradiated vessels in each subgroup.

**Figure 7:**
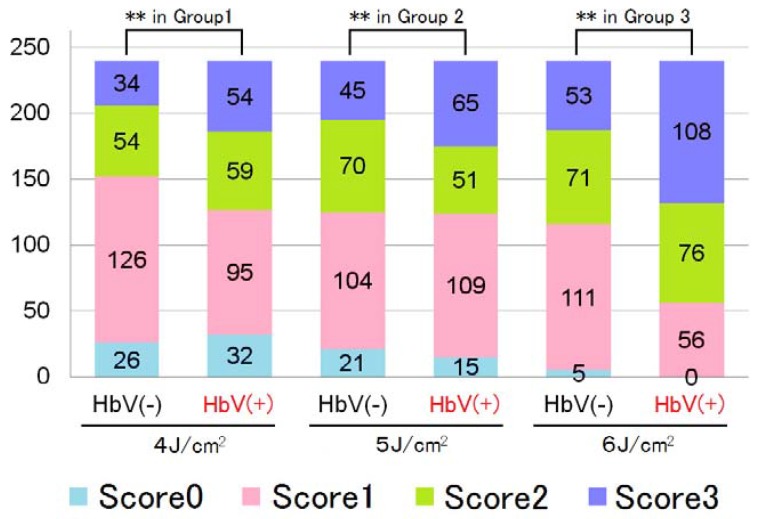
Distribution of the histopathological findings scores after dye laser irradiation at three difference fluences in the chicken wattle subgroups with and without the administration of hemoglobin vesicles (Hb-Vs). ** *P* < 0.01 for the difference from the Hb-V(-) group (chi-square test). We irradiated a laser beam by a V-beam dye laser (Candera Corp., California, USA; wavelength, 595 nm; pulse width, 0.45–40 ms). No cooling device was used to protect the skin surface.

The distribution of these histopathological findings scores is presented in Figure 7 [1]. Significant differences were found between the intervention and control subgroups in all three fluence groups (*P* < 0.01). This demonstrated that the severity of vascular destruction varied according to both the parameters of the laser output and whether an Hb-V injection had been administered.

## 6. Discussion

Treating PWSs remains challenging for most plastic surgeons and dermatologists because most PWSs cannot be completely cleared even after many sessions of treatment with a pulse dye laser, the treatment of choice [5,7]. Finding ways to enhance the efficacy of treatment is key [5,7,10,11,12]. Here, we have proposed a potential method to increase the clearance rate by using Hb-Vs to expand the capillaries and increase the amount of Hb in them, and thus to enhance the photothermal effect and thermal dispersion of dye laser treatment [1,2].

The primary finding of our study was that Hb-Vs exhibited the same response to the dye laser as human RBCs [1]. The Hb-V is a reliable chromophore that exhibits a photothermal effect resulting in the local concentration of thermal energy. In vivo, an intravenous injection of Hb-Vs at one-tenth of the circulating blood volume produced an increase of intravascular volume and microvessel dilatation [1]. Furthermore, the Fåhræus effect resulted in the Hb-Vs flowing near the vessel wall, resulting in a relative increase in the concentration of Hb-Vs in small vessels [3]. In this manner, vascular endothelial cells can effectively be exposed to thermal energy by administering an Hb-V intravenous injection before laser irradiation. Hb-Vs could contribute to the effects not only of dye lasers, but also of other laser treatments in which RBCs assume the role of the absorber or heater.

Laser treatment for PWSs with Hb-V has some advantages compared with the use of other chromophores such as a self-blood transfusion. Self-blood transfusion is conducted at the bedside under stringent controls in order to maintain the freshness of the blood and to prevent a mismatch between the patient and the blood formulation [22]. The use of Hb-Vs presents no such difficulties. In pediatric cases, anemia after the blood has been drawn may be a problem that has to be addressed. Besides these difficulties, self-blood transfusion does not have the same advantage in the size of chromophore as Hb-Vs, which are so small that they flow between the red blood cells in microvessels [16].

Laser treatment for PWSs using Hb-Vs also has some advantages compared with other chromophores, such as chemical photosensitizers. Because Hb-Vs do not leak from blood vessels into the dermis, they do not result in solar photosensitivity. In addition, the Hb-Vs and RBCs cooperate to produce thermal energy quickly. Hb-Vs can work better than chemical photosensitizers in combination with the existing light therapy equipment used worldwide, and there is no need to buy new, expensive equipment. Physicians can utilize their experience of PWS treatment that has been in practice over 30 years. These attributes of the Hb-V make it a suitable therapy method for non-malignant disorders and for pediatric patients, and its use brings economic benefits.

## 7. Conclusions

In the treatment of PWSs with light equipment, Hb-Vs fulfill the same function as human RBCs, providing a photosensitizer effect that destroys the vessel wall. Furthermore, Hb-Vs are easier for experienced physicians to use than other chemical photosensitizers and bring benefits in medical safety and economy.

## Figures and Tables

**Table 1:** Scores for the histopathological findings [1].

Histological Findings Score	Observed Phenomenon
0	No significant change
1	Agglutination or denucleation of RBCs
2	Injury to the vascular wall
3	Perivascular hemorrhage
